# Next-Generation Sequencing Technologies for Early-Stage Cutaneous T-Cell Lymphoma

**DOI:** 10.3389/fmed.2019.00181

**Published:** 2019-08-13

**Authors:** Kazuyasu Fujii, Takuro Kanekura

**Affiliations:** Department of Dermatology, Kagoshima University Graduate School of Medical and Dental Sciences, Kagoshima, Japan

**Keywords:** mycosis fungoides, early stage, T-cell receptor, rearrangement, next-generation sequencing

## Abstract

The diagnosis of early stage cutaneous T-cell lymphoma is often difficult, particularly in mycosis fungoides (MF), because the clinical presentation, histological findings, and laboratory findings of MF resemble those of inflammatory skin diseases such as atopic dermatitis, psoriasis, and parapsoriasis en plaque. Furthermore, MF sometimes occurs with or after these inflammatory skin diseases. The current diagnostic criteria heavily rely on clinical impressions along with assessments of T cell clonality. To make a diagnosis of early-stage MF, the detection of a malignant clone is critical. T cell receptor (TCR) gene rearrangements have been detected by southern blotting or polymerase chain reaction for this purpose, but the results of these methods are insufficient. High-throughput TCR sequencing has provided insights into the complexities of the immune repertoire. Accordingly, his technique is more sensitive and specific than current methods, making it useful for the detection of early lesions and monitoring responses to therapy.

## Introduction

Cutaneous T cell lymphomas (CTCLs) comprise a heterogeneous class of non-Hodgkin lymphomas that are derived from skin-tropic T cells. Mycosis fungoides (MF), the most prevalent type of primary CTCL, accounts for almost half of all cases (1–3). MF is clinically characterized by erythematous patches, plaques, or skin tumors, and is can be associated with lymph node, blood, and internal organ involvement. More than two-thirds of MF patients are in early stage at first presentation (3–5). MF often starts as an unspecific erythema, similar to many inflammatory skin diseases.

Histopathologically, MF can be characterized by the epidermotropic proliferation of small- to medium-sized pleomorphic lymphocytes forming intraepidermal collections, also called Pautrier's microabscesses. This microabscess is considered the histopathological hallmark of disease, but it is only observed in <20% of early MF cases (6). These microabscesses are also usually recognized as epidermotropic atypical lymphocyte infiltration without spongiosis, although spongiotic variants of MF have been reported (7, 8). Morphologic characterization of early-stage MF might show non-specific findings (9), because skin lesions are infiltrated by large numbers of non-malignant memory T cells, often making it impossible to distinguish malignant T cell clones from activated benign infiltrating T cells based on histopathology (6). Clinical and histopathological algorithms have been developed to aid early diagnosis (10), but the specificity and sensitivity of these algorithms for early diagnosis in individual patients are by no means established. A definitive diagnosis can only be made based on careful clinicopathological correlations (9).

Early stages of MF can resemble benign inflammatory skin disorders (11, 12) like chronic dermatitis including atopic dermatitis (AD), psoriasis, and parapsoriasis en plaque (PEP), among others. AD is a common chronic inflammatory skin disorder that has a T-helper (Th) 2 type-dominant phenotype, skin-barrier dysfunction, and pruritus (13). In contrast AD is an inflammatory disorder, and its pathophysiology is similar to that of AD. Mycosis fungoides, characterized as a Th2-type disease (14, 15), is frequently linked to eosinophilia and high serum immunoglobulin E levels. Although affected skin and peripheral blood T-cells express a Th1 cytokine profile during early-stage MF (16, 17), chemokines expressed in MF lesional skin, such as CCL17, CCL11, and CCL26, are supposed to induce a Th2 milieu in MF (18).

Barrier dysfunction is also observed in MF (19). Lower levels of skin moisture, with increased transepidermal water loss, have been observed in the lesional skin of CTCL, compared to that in normal skin. CTCL lesional skin also displays decreased levels of *filaggrin* and *loricrin* mRNAs compared to those in normal skin, which has also been demonstrated for AD. Pruritus is often present in MF patients (20) and constitutes one of the most disturbing symptoms for patients (21). Therefore, it is occasionally difficult to clinically differentiate MF from AD. The coexistence of MF and AD in patients was also reported in several studies (22, 23).

Psoriasis is a common, chronic inflammatory skin disorder defined by thickened, red, scaly plaques with systemic inflammation. The relationship between MF and psoriasis is sometimes difficult to determine, as there is often significant overlap in terms of pathological and clinical observations, particularly in early stages (24). Psoriasis and MF have the abnormal function of T cells as a common symptom. Psoriasis was recognized as a Th1 disease, although recent data suggest that it might be a Th17 disease (25). MF during preliminary stages also exhibits a Th1 phenotype (26); moreover, Krejsgaard et al. (27) reported that malignant T cells from CTCL lesions produce IL-17. Therefore, many early MF cases are misdiagnosed as psoriasis, whereas another group of cases occur in which the two diseases coexist and/or psoriasis develops into MF. The prevalence of psoriasis among patients with MF was found to be higher than that estimated for the general population (24, 28) and patients with psoriasis have an elevated risk of lymphomas including CTCL (29, 30). In addition to the common symptoms, immunosuppressive agents might also promote MF development in patients with psoriasis (31).

PEP is a chronic, inflammatory skin disorder, closely resembling early-stage MF. Clinically, PEP consists of persistent, scaly, well-demarcated erythematous lesions. Pathologically, it is associated with superficial lymphocyte infiltration with various degree of epidermotropism (32). More than 30% patients with large plaque parapsoriasis develop pathologically-confirmed MF (32), and therefore, PEP is often an early manifestation of MF. However, the individual clinical course might determine the difference between early-stage MF and PEP.

Because of difficulties associated with differential diagnosis, MF often remains undiagnosed for years. Accordingly, the average time from the appearance of lesions to a definitive diagnosis was to be estimated 3–6 years (33, 34).

## T-cell Receptor Clonality is an Important Criterion in MF Diagnosis

Diagnosing T-cell malignancies is often hampered by difficulties in distinguishing neoplastic T cells from reactive T cells based on conventional morphological and immunopathological criteria (35). Skin lesions of MF patients are infiltrated by many non-malignant memory T cells, and thus, it is often impossible to distinguish a malignant T cell clone from activated, benign, infiltrating T cells by histopathology, particularly for early-stage lesions (10).

**Figure 1:**
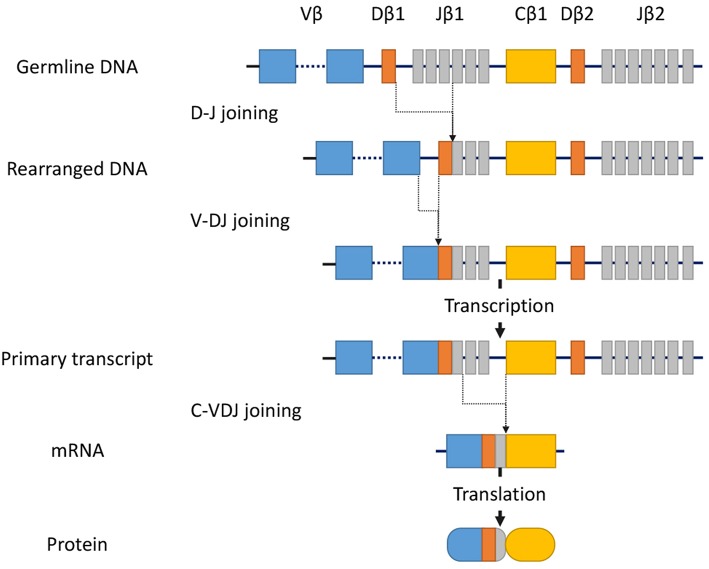
Somatic recombination of the *TCR*β gene. Rearrangement begins with D-J recombination followed by subsequent V-DJ recombination. The segments recombine randomly to generate TCR diversity. After transcription, intervening sequences are spliced, generating the *TCR*β chain transcript with V, D, J, and C region segments. Finally, transcripts are translated into protein. In contrast, the *TCR*α gene lacks the D-segment, and its rearrangement starts with V-J recombination.

T-cell receptor (TCR) gene configurations are thought to be the most promising marker to identify malignant T-cell proliferation (36). TCRs are cell-surface protein heterodimers that are expressed on T cells and comprise either α and β chains or γ and δ chains. Immature T-cells rearrange their TCR genes during maturation in the thymus, then mature as either αβ or γδ lineage T-cells (37). TCRs are unique to individual T cell clones. Malignant cells in MF express αβ TCRs. The gene segments that encode variable (V), diversity (D) (β and δ chains only), joining (J), and constant (C) domains of the TCR protein exist as multiple unique sets (38). Since TCR genes are rearranged during thymic T-cell development (Figure 1) but not in mature T-cells, a peripheral T-cell lymphoma clone of malignant cells should only have a single TCR gene sequence. TCR genes are rearranged polyclonally in normal and reactively proliferating T cells as rearrangements are random, whereas neoplastic cells contain identically-rearranged TCR genes. Similarly, molecular analysis of such rearrangements can be useful to differentiate MF from benign skin conditions (39). Half of patients with large plaque parapsoriasis were reported to have TCR monoclonality in the lesional skin (40), and thus, the detection of monoclonality in a skin lesion generally suggests CTCL including MF, rather than inflammatory skin disorders.

## Conventional Methods for Detecting T-cell Clonality

Initially, Southern blotting was used to determine TCR gene rearrangement clonality (41). This technique can detect clonal T-cell populations in most T-cell lymphomas without prior amplification, but has several disadvantages including low sensitivity and the requirement for large amounts of fresh frozen tissue. Therefore, since the effectiveness of Southern blotting in the diagnostic work-up of MF was reported (42–44) in the early 1990s, more sensitive polymerase chain reaction (PCR)-based methods have been developed. PCR amplification of rearranged TCR gamma genes using genomic DNA as the template was reported to permit the detection of clonal T cells with a sensitivity of 0.1–1% from a background population of polyclonal T-cells (45). Conventional agarose gel electrophoresis of the PCR products often fails to reliably differentiate polyclonal from monoclonal TCR junctions (46), because the narrow size range of the PCR products makes multiple bands appear as single bands. Therefore, PCR amplification with subsequent denaturing gradient polyacrylamide gel electrophoresis and gel scanning (47, 48) or the Biomed GeneScan analysis of flat or capillary polyacrylamide gels (49, 50) has been used as a diagnostic assay for clonality in CTCL patients.

Despite these technical advances, current methods for TCR clonality are still sometimes insufficient for a definitive diagnosis, particularly at early stages, because early lesions often do not contain sufficient numbers of clonal T-cells (51–53). These non-quantitative tests have significant false negative and positive rates for MF (50, 54) and are particularly unreliable for early-stage MF (55).

## High-Throughput Sequencing Technologies for Diagnosing MF

Recent improvements in assays to assess T cell clonality have been achieved based on next-generation high-throughput sequencing (NGS) technologies. By sequencing the third complementarity-determining regions (CDR3s) of genes encoding TCRβ and TCRγ, the number of individual T cell clones present in a sample, the relative proportions of specific clones, and the CDR3 region sequences of each clone can be quantified (56, 57). Likewise, NGS represents a superior method to diagnose CTCL through the precise identification of malignant T cell clones (58–60). This technique is more sensitive than previous techniques for the detection of clonality (59, 60). Further, NGS-based methods allow the clinician to follow specific clones when monitoring disease recurrence and progression (60). Furthermore, TCR sequencing has clarified that neoplastic cells in some MF lesions might be as few as 1% of the total population of T cells (59). These data clearly explain the difficulties encountered in the histopathological assessment of early-stage MF. In contrast, the frequencies of the most dominant T cell clones range from 1 to 10% in most cases of inflammatory skin disorders such as psoriasis and eczematous dermatitis, among others, whereas the frequency of the most dominant T cell clones adjusted by total nucleated cells could distinguish MF from inflammatory skin disorders (59). Therefore, PEP often demonstrates TCR rearrangement, and NGS-based TCR gene analysis might overcome difficulties in distinguishing PEP and early-stage MF T-cell repertoires.

Most MF cases present as early-stage, typically with a chronic, indolent clinical course. Greater than 80% of patients with early-stage disease will present with an indolent life-long course, free of disease progression, independent of the treatment modality (5). For many years, most patients will also exhibit short-term clinical response associated with recurrent disease, as well as a normal life expectancy in the majority of cases. Furthermore, the limited efficacy associated with chemotherapy has been discussed in retrospective studies (61, 62), making it clear that potentially toxic and aggressive therapies should be avoided (63, 64). However, a small number of early-stage cases will progress. Since advanced-stage patients have poor prognoses, the early identification of high-risk subpopulations is important.

Using NGS technologies, de Masson et al. (55) demonstrated that an enhanced proportion of a malignant T cell clone in the skin is strongly correlated with reductions in progression-free and overall survival for patients with CTCL, and particularly for patients with early-stage MF with a T2 distribution. Further, based on high throughput DNA sequencing of the TCRβ gene, a tumor clone frequency of >25% was found to be a strong predictor of disease progression and poor survival for MF patients with disease limited to the skin.

In summary, evidence for TCR clonality from any method is strong evidence for malignancy. However, it is not conclusive, because benign conditions have also been associated with clonal T-cell populations, such as reactive or autoimmune conditions (65, 66).

## Conclusion

NGS can be used to assess TCR clonality with superior sensitivity compared to current methods and is useful to diagnose early stage MF. Moreover, this technique permits the tracking of specific clones across different time points or in multiple lesions for a more accurate diagnosis of MF recurrence or progression (55, 59, 60).

## Author Contributions

KF conceived the concept and wrote the manuscript. TK edited and improved the manuscript.

### Conflict of Interest Statement

The authors declare that the research was conducted in the absence of any commercial or financial relationships that could be construed as a potential conflict of interest.
